# Sampling strategies to evaluate the prognostic value of a new biomarker on a time-to-event end-point

**DOI:** 10.1186/s12874-021-01283-0

**Published:** 2021-04-30

**Authors:** Francesca Graziano, Maria Grazia Valsecchi, Paola Rebora

**Affiliations:** BICOCCA BIOINFORMATICS BIOSTATISTICS AND BIOIMAGING CENTRE-B4, School of Medicine and Surgery, University of Milano – Bicocca, Via Cadore 48, 20900 Monza, Italy

**Keywords:** Case-control design, Cohort studies, Power, Two-phase sampling, Weighted cox model

## Abstract

**Background:**

The availability of large epidemiological or clinical data storing biological samples allow to study the prognostic value of novel biomarkers, but efficient designs are needed to select a subsample on which to measure them, for parsimony and economical reasons. Two-phase stratified sampling is a flexible approach to perform such sub-sampling, but literature on stratification variables to be used in the sampling and power evaluation is lacking especially for survival data.

**Methods:**

We compared the performance of different sampling designs to assess the prognostic value of a new biomarker on a time-to-event endpoint, applying a Cox model weighted by the inverse of the empirical inclusion probability.

**Results:**

Our simulation results suggest that case-control stratified (or post stratified) by a surrogate variable of the marker can yield higher performances than simple random, probability proportional to size, and case-control sampling. In the presence of high censoring rate, results showed an advantage of nested case-control and counter-matching designs in term of design effect, although the use of a fixed ratio between cases and controls might be disadvantageous. On real data on childhood acute lymphoblastic leukemia, we found that optimal sampling using pilot data is greatly efficient.

**Conclusions:**

Our study suggests that, in our sample, case-control stratified by surrogate and nested case-control yield estimates and power comparable to estimates obtained in the full cohort while strongly decreasing the number of patients required. We recommend to plan the sample size and using sampling designs for exploration of novel biomarker in clinical cohort data.

**Supplementary Information:**

The online version contains supplementary material available at 10.1186/s12874-021-01283-0.

## Background

In the past decades, there has been a growing number of epidemiological [1–3] and longitudinal studies storing biological samples [4] to allow retrospective evaluation of new research questions, such as evaluating the prognostic value of new biomarkers. This approach is convenient, as it significantly reduces the time needed for the study. However, the analysis of novel biomarkers can be expensive. Sub-sampling strategies result in considerable cost savings and parsimonious use of biological specimens, by restricting data extraction to an informative subgroup of the original sample. Unbiased and more precise results can be obtained if the subgroup is carefully sampled rather than chosen at random [5, 6].

Two-phase sampling is a general approach to perform such sub-sampling, including case-control and case-cohort designs [7, 8]. This approach considers the entire cohort as the phase I sample from the population of interest. In the phase II, subsamples are drawn from the cohort to measure additional information, such as new biomarkers of interest [9]. An optimal sampling strategy was proposed for stratified two-stage studies with binary outcome, however it needs the availability of pilot data on the biomarker of interest that are not always available. Moreover, there is no literature on criteria for the choice of the stratification variables and on the case of time-to-event outcome.

The sample size of retrospective studies is often planned considering budget constrains rather than a proper evaluation of the statistical power [10], also due to the lack of methodologies for power calculation in this setting. Cai and Zeng [11] focused on power in case–cohort design without any stratification; Haneuse et al. [12, 13] focused on binary outcomes, but a general strategy for power evaluation is missing for survival data.

In this study we compared different sampling designs in the two-phase setting, where the aim is to assess the prognostic value of a new biomarker on a time-to-event end-point, and provided a simulation tool to estimate power. In particular, we focused on the sampling design of the sub-cohort on which to measure the new biomarker. The principal goal was to investigate the performance of different sampling designs and the contribution of stratification variables available in the full cohort (e.g. surrogate, risk factor and confounder). We chose the two-phase setting as a general framework in which it is possible to include and compare different types of common designs. We performed a power evaluation varying the sub-cohort sample size. We used real data from a randomized trial in childhood acute lymphoblastic leukemia (ALL). Briefly, this study was performed to evaluate the role of different genetic polymorphisms on treatment failure due to relapse [14, 15]. Clinical data and other information were available for the whole trial cohort and biological samples were stored at diagnosis. The genetic polymorphisms were retrospectively evaluated on these specimens using a two-phase design.

## Methods

### Notation settings

A survival analysis notation is used as the focus is on a time-to-event end-point. Let *T*_*i*_ be the failure time and *C*_*i*_ the censoring time of subject *i* (*i* = 1…*N*) in a cohort (phase I) of size N followed-up to time τ. *T*_*i*_ and *C*_*i*_ are assumed to be independent, *T* ⊥ *C*, indicating a non-informative right censoring. Administrative censoring is set at the end of follow-up time τ. Let *h*_*i*_(*t*) be the hazard rate for the *i* th individual . The hazard function, modelled using the Cox proportional hazards model, is equal to *h*_*i*_(*t*) = *h*_0_(*t*) exp(*βX*_*i*_) where *h*_0_(*t*) is the baseline hazard, *X*_*i*_ the vector of the explanatory variables for individual *i* and *β* ’s the corresponding regression coefficients. The classical approach for estimating *β* is to maximize the partial likelihood [16]. Suppose that the biomarker of interest, i.e. *X*_*BM*_, is measured only for a subset n < N of subjects drawn from the phase I data and let ξ_*i*_ indicate whether subject *i* is selected into this subset. We will refer to the $n={\sum}_{i=1}^N{\upxi}_i$ subjects as the phase II sample. Let π_*i*_ = *P*(ξ_*i*_ = 1 ∣ *X*_*i*_, ∆_*i*_, *Z*_*i*_) being the inclusion probability of subject *i* for the phase II sample, conditional on being selected at phase I. In a simple random sample this probability is equal for every subject (*π* = *n*/*N*). In a stratified sampling, the inclusion probability is common for all subjects in the same stratum and differs between strata. In particular, it is usually higher for the more informative strata (e.g. strata including subjects with the event of interest, as in case-control studies).

### Simulation context

#### Phase I sample

To mimic a realistic context, we explore the variables that well represent the majority of data usually available in practice, even though in a simplified setting for simulation.

**Fig. 1:**
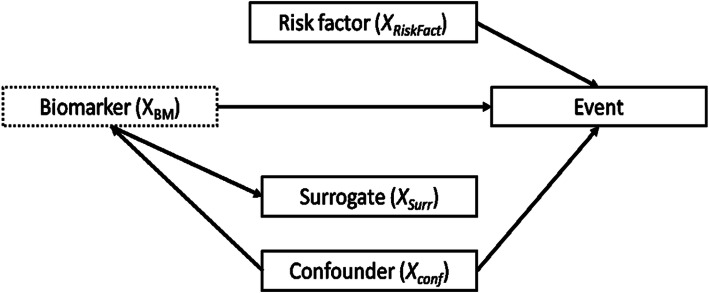
Causal diagram where the variables in the boxes are connected each other through the black arrows, denoting association. The dashed line box indicates a variable measured only in the sub-cohort

We hypothesized a cohort of subjects of size N (i.e. clinical trial cohort, register, clinical cohort) followed up to time τ, in which we aim to evaluate the prognostic value of a new biomarker (*X*_*BM*_) on a time-to-event end-point (*T*) in the presence of a possible confounder (*X*_*Conf*_), a risk factor (*X*_*Risk Fact*_) and a possible auxiliary/surrogate variable (*X*_*Surr*_) of the marker of interest. To describe and illustrate relationships between these, a Directed Acyclic Graph (DAG) was displayed in Fig. 1. In particular, we assumed the confounder to have an impact on both the biomarker and the event of interest, the risk factor to be associated only with the event of interest, and, finally, the surrogate to be associated only with the biomarker.

#### Phase II sample

We assumed that the risk factor, the confounder and the surrogate variables are known for all subjects in the phase I (N), while the biomarker (*X*_*BM*_) is measured only on the subset of n individuals (phase II sample).

To sample the subset of subjects from phase I (N), a stratified two-phase sampling approach was used. Strata were defined using the following variables: event or event and risk factor or event and confounder or event and surrogate.

By note, in this work, we consider only sampling done at the end of the follow-up (τ) where subjects who developed the event during the follow-up are defined as cases and subjects event-free at time τ as controls.

The sample size of the phase II is fixed (n), but the sampling probabilities depend on different designs, as described below:

(i) Simple Random Sample (SRS) in which all possible subsamples have an equal probability to be chosen.
(ii) Probability Proportional to Size (PPS) is referred to a stratified sample with proportional allocation. The units are selected with probabilities proportional to stratum’s size. Thus, the size for each stratum in the phase II is given by the total size of the stratum in the original cohort multiplied by n/N [17].
(iii) Case-Control (CC) is performed by separately sampling cases and controls [18]. As we aimed to compare different sampling strategies with a fixed sample size, we did not necessarily select all cases from the full cohort as often done. We fixed a total sample size (n) and selected an equal number of cases (n/2) and controls (n/2). We also considered stratified CC by using the variables available in phase I (see Fig. 1): separated simple random sampling was performed in each stratum. A balance design was considered [19].
(iv) Nested case-control (NCC) can be considered as a particular case of case-control designs in which controls are randomly selected from the set of subjects event-free at the time of event occurrence on the cases [20–22]. Sampling probabilities for controls were derived by Samuelsen [23], while for cases they were equal to 1 if the phase II sample size n was at least twice the total number of events in the entire cohort (${\sum}_{i=1}^N{\Delta }_i\Big)$ and equal to ${\pi}_i=\left(n/2\right)/\left({\sum}_{i=1}^N{\Delta }_i\right)$ otherwise.
(v) Counter matching (CM) is an alternative stratified version of the NCC. In this design, the selection of controls is conducted by sampling from the set at risk in the opposite stratum at the time of event on the case. Inclusion probabilities for controls within strata were derived by Samuelsen [24] while for cases, *π*_*i*_ was derived as in NCC design. As the aim is to maximize the “discordance” of exposure within case-controls sets [25–27], the variables used to define strata must be a proxy for the variables of interest, thus we used the surrogate variable *X*_*Surr*_ for this design.

**Fig. 2:**
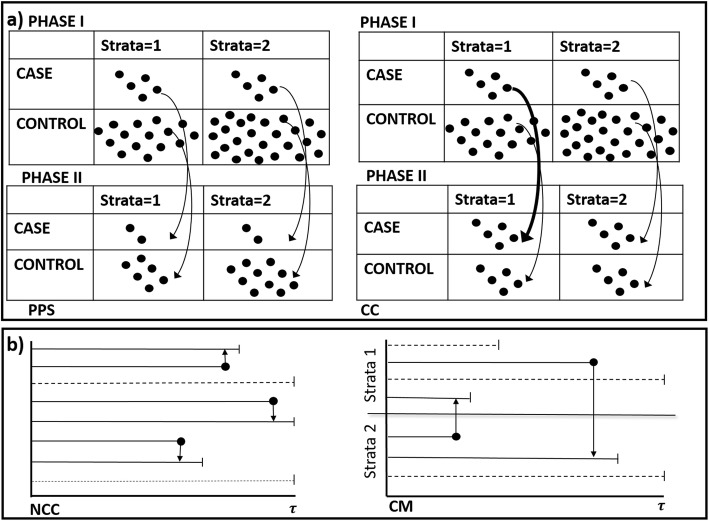
Probability Proportional to size (PPS) and Case-Control (CC) sampling from phase I cohort are shown in the upper part (**a**), left and right, respectively. Dots represents individuals in the strata (case or control and strata = 1 or strata = 2). Arrows correspond to the sampling from phase I to phase II. The number of sampled individuals in each stratum (phase II) depends on the sampling design. Nested Case-Control (NCC) and Counter-matching (CM) sampling are shown below in the figure (**b**). The lines represent the follow-up over which individuals are observed and the solid lines represented the sampled subjects. Black dot symbol represents the occurrence of an event and the arrow indicated the corresponding sampled control. For NCC, sampling is conducted in the same stratum and for CM, cases are matched with controls from the opposite stratum

Figure 2 illustrates an example of each sampling design method described above. Specifically, in the upper part of the figure we displayed PPS and CC considering a stratification for a binary variable; in the lower part NCC and CM designs are displayed. By note, in NCC and CM designs we considered one control selected for each case.

### Evaluation of biomarker impact on the event

The following Cox model was applied to assess the influence of the biomarker on hazard of the event adjusting for the confounder variable *X*_*Conf*_ (following the minimal set of adjustment suggested in Fig. 1):

1 $$ {h}_i(t)={h}_0(t)\ \exp \left({\beta}_{BM}{X_{BM}}_i+{\beta}_{Conf}{X_{Conf}}_i\right) $$

where *β*_*BM*_ and *β*_*Conf*_ represent the regression coefficients of the biomarker and confounder, respectively. Given the availability of the biomarker only for the sub-cohort (phase II), we applied a weighted Cox model, in which regression coefficients are estimated by maximizing the partial likelihood weighted by the inverse of the empirical inclusion probability ($$ {w}_i=\raisebox{1ex}{$1$}\!\left/ \!\raisebox{-1ex}{${\uppi}_i$}\right. $$) that accounts for the specific sampling design [6, 28, 29]. In SRS, CC and PPS designs [17, 30] empirical inclusion probabilities (π_*i*_) were calculated using a standard approach implemented in the “twophase function” in the survey package. Instead, π_*i*_ ’s were calculated following Samuelsen [23] for NCC and following Rivera for CM [25].

As surrogate variables are rarely available for new biomarkers at the design stage, we considered also a setting with post-stratification for surrogate variables, mimicking a possible situation in which the surrogate variable is identified only after sampling, as this might still be advantageous [31]. In order to estimate this advantage, we performed a classical CC sampling design and then we ran a weighted Cox model post-stratifying for the surrogate variable [8].

### Simulations parameters

The performance of the different designs was investigated through simulations. The number of simulations needed to guarantee robust results was calculated following Burton et al. [32]. It was set at B = 2000 assuming a level of accuracy equal to 0.0046 and a variance of *X*_*BM*_ regression coefficient estimate equal to 0.011 with a 5% significance level. To generate the hypothetical cohort described above, for each scenario we drew B = 2000 random phase I samples of *N* = 2000 subjects.

We started by simulating the confounder variable as a dichotomous variable with *P*(*X*_*Conf*_ = 1) = 0.5; the biomarker was simulated by a binomial distribution with *P*(*X*_*BM*_ = 1| *X*_*Conf*_) = exp(*a* + *b* ∗ *X*_*conf*_)/(1 + exp(*a* + *b* ∗ *X*_*conf*_)) resulting in a prevalence in the entire cohort of nearly 25% (a = − 2 an b = 1.7) and ~5% (a = − 4 and b = 1.5) for common and rare biomarker, respectively. The surrogate/auxiliary variable, with *X*_*BM*_ as gold-standard, was simulated as *P*(*X*_*Surr*_ = 1| *X*_*BM*_) = exp(*c* + *d* ∗ *X*_*BM*_)/(1 + exp(*c* + *d* ∗ *X*_*BM*_)). In order to cover different levels of accuracy of the surrogate in “predicting” the value of the biomarker, we set different values of parameters *c* and *d* (see the Additional file Table S1 for details) resulting in specificity (*P*(*X*_*Surr*_ = 0| *X*_*BM*_ = 0)) and sensitivity (*P*(*X*_*Surr*_ = 1| *X*_*BM*_ = 1)) values ranging between 70 to 90%. Finally, an additional binary risk factor *X*_*Risk factor*_ was generated with a probability of *P*(*X*_*Risk factor*_ = 1) = 0.4.

The time-to-event endpoint was generated [32, 33] from a Weibull hazard model as $$ T={\left(- logU/\lambda \exp \left({\beta}^{\prime }X\right)\right)}^{\raisebox{1ex}{$1$}\!\left/ \!\raisebox{-1ex}{$p$}\right.} $$, where *p* = 0.9, *λ* =0.1, with U following a uniform distribution on the interval from 0 to 1 and with the matrix of covariates X including the biomarker value (*X*_*BM*_), the risk factor (*X*_*Risk Factor*_) and the confounder (*X*_*Conf*_). A random right censoring time was generated from an exponential distribution and three different censoring rates were considered (*ρ* equal to 0, 0.1, 0.4) to yield 0, 15 and 50% subjects censored at the end of follow-up time τ. Minimum between time-to-event *T*_*i*_ and censoring *C*_*i*_ (*Z*_*i*_ = min(*T*_*i*_, *C*_*i*_)) was calculated, with ∆_*i*_ = *I*(*T*_*i*_ < *C*_*i*_). Administrative censoring was set at τ =2. This setting resulted in an average of 500 events for each phase I dataset at the end of follow-up. The values for the regression coefficients (*β*) and baseline hazard were chosen to mimic the observed values in ALL data [15, 34]. Details of all specific parameters were reported in the Additional file Table S1.

The sampling design scheme for the phase II (size n) was illustrated in the paragraph Phase II Sample in section Simulation context. In particular, we performed SRS, PPS and CC (the last two stratified by event or event and risk factor or event and confounder or event and surrogate) and, finally, NCC and CM.

Information on *X*_*BM*_ was disregarded for subjects not included in the phase II sample and a weighted Cox model was applied to estimate *β*_*BM*_ as described in Evaluation of Biomarker impact on the event section.

The performance of the estimate of *β*_*BM*_ over the B simulations has been assessed by the following measures [32]:

(i) Bias, given by = ${\overline{\hat{\beta}}}_{BM}-{\beta}_{BM}$, where ${\overline{\hat{\beta}}}_{BM}$ = $\frac{\sum_{i=1}^B{\hat{\beta}}_{iBM}}{B}$,
(ii) $SE\left({\hat{\beta}}_{BM}\right),$ the empirical Standard Error (SE) of *β*_*BM*_ over all simulations,
(iii) Design effect, defined as the ratio between the estimated variance of *β*_*BM*_ in each sampling design by the one in SRS [35],
(iv) Mean Square Error, MSE, given by ${\left({\overline{\hat{\beta}}}_{BM}-{\beta}_{BM}\right)}^2+{\left( SE\left({\hat{\beta}}_{BM}\right)\right)}^2$,
(v) Coverage of the 95% confidence interval (CI) of *β*_*BM*_ and 95%CI length,
(vi) Power, number of times in which the null hypothesis (*β*_*BM*_ = 0) was rejected by the Wald test at 5% significance level in the weighted Cox regression model.

All analyses were performed using R software (version 3.5.2) [36].

## Results

### Design comparison

General results considering both a common (~ 25% prevalence) and rare biomarker (~ 5% prevalence) are shown in Table 1a-b, respectively, under three censoring levels (absent, low and high). Overall, the simulations showed that the *β*_*BM*_ was estimated without any noticeable bias for all designs. The standardized bias was always lower than 5% and the distribution of ${\hat{\beta}}_{BM}$ was symmetric for all sampling designs (Additional file Figure S1).

**Table 1:** Bias, empirical standard error, mean square error, power and design effect of the biomarker regression coefficient estimate (${\hat{\beta}}_{BM}$) for the full cohort and different sampling designs. Accuracy of surrogate: sensitivity (i.e. probability of having a positive surrogate if the biomarker is positive) = 0.7 and specificity (i.e. probability of having a negative surrogate if the biomarker is negative) = 0.7, biomarker common (a) and rare (b)

Sampling design	Stratification variable	n*	Bias			SE empirical			MSE			Power (%)			Design effect		
			Censoring rate			Censoring rate			Censoring rate			Censoring rate			Censoring rate		
			0	0.1	0.4	0	0.1	0.4	0	0.1	0.4	0	0.1	0.4	0	0.1	0.4
**a)**																	
Full cohort	–	2000	0.008	−0.015	0.009	0.093	0.095	0.112	0.009	0.009	0.013	99	97	95	–	–	–
1. SRS	–	600	0.004	−0.013	0.006	0.182	0.187	0.206	0.033	0.035	0.042	64	58	53	–	–	–
2. PPS	Event	599	0.007	−0.015	0.007	0.173	0.180	0.199	0.029	0.033	0.039	65	58	54	1.003	1.003	1.005
2a. PPS	Event; Risk factor	598	0.008	−0.016	0.004	0.172	0.175	0.205	0.029	0.031	0.042	65	58	52	1.002	1.003	1.002
2b. PPS	Event; Confounder	598	0.003	−0.015	0.002	0.174	0.179	0.203	0.030	0.032	0.041	65	57	51	0.999	1.002	1.000
2c. PPS	Event; Surrogate	598	0.007	−0.013	0.013	0.161	0.171	0.190	0.026	0.029	0.036	69	64	57	1.106	1.129	1.104
3. CC	Event	600	0.011	−0.008	0.019	0.159	0.158	0.179	0.025	0.025	0.032	74	68	67	1.179	1.219	1.352
3a. CC	Event; Risk factor	600	0.010	−0.009	0.008	0.162	0.166	0.182	0.026	0.028	0.033	72	65	62	1.139	1.176	1.307
3b. CC	Event; Confounder	600	0.012	−0.015	0.010	0.162	0.161	0.175	0.026	0.026	0.031	73	65	66	1.182	1.187	1.354
3c. CC	Event; Surrogate	600	0.008	−0.016	0.012	0.148	0.153	0.170	0.022	0.024	0.029	76	71	69	1.334	1.363	1.495
4. NCC	Event	550	0.008	−0.018	0.014	0.169	0.165	0.175	0.029	0.028	0.031	68	63	67	1.066	1.144	1.378
5. CM	Event; Surrogate	546	−0.044	−0.058	− 0.009	0.151	0.153	0.165	0.025	0.027	0.027	67	61	67	1.379	1.395	1.536
**b)**																	
Full cohort	–	2000	−0.028	−0.004	−0.023	0.193	0.199	0.225	0.038	0.039	0.052	52	53	41	–	–	–
1. SRS	–	600	−0.045	− 0.041	−0.093	0.376	0.392	0.802	0.144	0.156	0.652	23	23	19	–	–	–
2 PPS	Event	599	−0.051	−0.023	−0.060	0.380	0.394	0.453	0.147	0.155	0.209	25	24	19	1.079	1.131	1.139
2a. PPS	Event; Risk factor	598	−0.048	−0.039	− 0.081	0.372	0.393	0.721	0.141	0.156	0.526	22	24	21	1.085	1.109	1.145
2b. PPS	Event; Confounder	598	−0.057	−0.033	−0.059	0.381	0.393	0.453	0.148	0.156	0.209	23	23	20	1.077	1.104	1.140
2c. PPS	Event; Surrogate	598	−0.055	−0.022	− 0.073	0.388	0.391	0.597	0.153	0.153	0.362	24	25	19	1.090	1.147	1.158
3. CC	Event	600	−0.003	0.021	0.003	0.332	0.339	0.368	0.110	0.116	0.135	24	26	22	1.257	1.317	1.509
3a. CC	Event; Risk factor	600	−0.011	0.015	0.006	0.345	0.357	0.384	0.119	0.127	0.148	23	24	21	1.193	1.283	1.418
3b. CC	Event; Confounder	600	−0.023	0.024	0.011	0.328	0.344	0.363	0.108	0.119	0.132	23	26	22	1.267	1.329	1.513
3c. CC	Event; Surrogate	565	−0.018	0.011	−0.010	0.311	0.313	0.345	0.097	0.098	0.119	26	29	24	1.419	1.607	1.757
4. NCC	Event	545	−0.020	0.024	−0.000	0.354	0.355	0.363	0.126	0.127	0.132	21	24	21	1.126	1.222	1.513
5. CM	Event; Surrogate	529	−0.057	−0.041	−0.028	0.311	0.315	0.341	0.099	0.101	0.117	26	27	25	1.541	1.679	1.823

Legend: *SRS* Simple Random Sample, *PPS* Probability Proportional to size, *CC* Case-Control, *NCC* Nested Case-Control, *CM* Counter-matching; n* mean sample size of the full cohort (first row) and of the phase II sample

As shown in Table 1a, PPS did not show much advantage compared to SRS design. The empirical Standard Error of SRS and PPS were about the same, indicating no gain in efficiency. We found a small but not relevant increase of power in PPS stratified by the surrogate (2c in Table 1a) compared with traditional PPS.

On the other hand, CC design improved power as compared with SRS reducing MSE and empirical Standard Error (for each scenario), with a further advantage when the surrogate variable was used for stratification (3c in Table 1a). The stratification for risk factor and confounder (3a and 3b in Table 1a) showed a slight loss of efficiency with respect to the classical CC (3 in Table 1a).

When matching on time, CM presented higher design effect and smaller confidence intervals with respect to CC stratified by the surrogate for any censoring rate. The best advantage of NCC and CM (4 and 5 in Table 1) as compared with CC, PPS, and SRS, in terms of Mean Square Error, empirical Standard Error and design effect, was obtained when censoring rate was higher (*ρ* = 0.4). Among all scenarios, the CC stratified by the surrogate showed the highest power. By note, in NCC and CM design, the actual sample size of the phase II was sligthly lower than the planned one (expected phase II *n* = 600, observed *n* = 550 and 546 for NCC and CM, respectvely, see Table 1) due to the possible resampling of controls.

Similar performance results were obtained when a rare exposure (~ 5%) was considered (Table 1b). In general, with a rare exposure, performance of NCC and CM, in term of bias, design effect and width of 95%CI, had an improvement with increasing censoring rates. The estimate of regression coefficient (${\hat{\beta}}_{BM}\Big)$, width and coverage of its 95% confidence interval considering common and rare biomarker are given as Additional file Table S2. Due to the presence of a slight bias (lower than 5%), the design effect was also calculated using the Mean Square Error, MSE [37, 38]. Results (showed in Additional file Table S4) were consistent.

As sensitivity analysis, we evaluated the performance of the different designs including in the weighted Cox model [1] not only the confounder but also the risk factor and surrogate variable. Results are presented in the Additional file (Table S3) and are consistent with previous results. Interestingly, when the weighted Cox model was adjusted also for the risk factor variable (Table S3a), *X*_*Risk Factor*_, there was an increase in power for all designs as compared with results of Table 1a. On the contrary, when the Cox model was adjusted for all variables available in our setting (i.e. confounder, *X*_*Conf*_, risk factor *X*_*Risk Factor*_ and surrogate, *X*_*Surr*_, see Table S3b), power decreased.

### Impact of surrogate

**Fig. 3:**
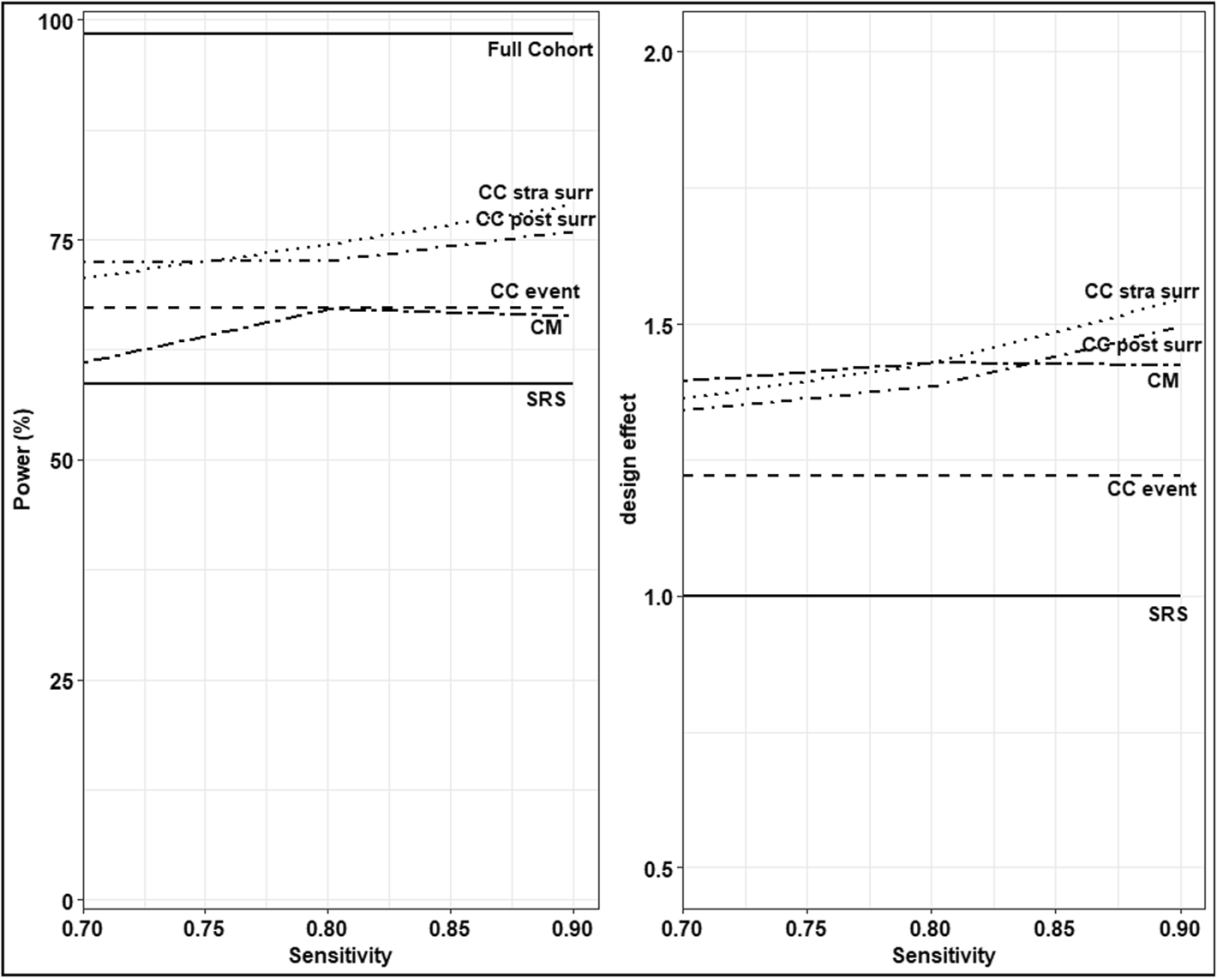
Power and design effect for different sensitivity levels (i.e. probability of having a positive surrogate if the biomarker is positive) of the surrogate variable. Scenario: specificity (i.e. probability of having a negative surrogate if the biomarker is negative) =0.7, censoring rate ρ = 0.1, hazard ratio of biomarker =1.5 and sample size of phase II (n) =600. Legend: CC stra surr (Case-Control stratified by surrogate), CC post surr (Case-Control post stratified by surrogate), CC event (Case-Control), CM (Counter-Matching) and SRS (simple random sampling)

In this section, we explore the impact of the accuracy level of the surrogate variable in the model performance. As expected, higher sensitivity increased power and design effect in the CC design stratified by the surrogate and in the CM design (Fig. 3). The post-stratification for the surrogate variable only in the analysis stage (surrogate not used as strata in the design) showed an advantage both in power and design effect as compared with CC design and a disadvantage as compared with CC design stratified by the surrogate. Its performance increased with increasing accuracy.

### Power evaluation

**Fig. 4:**
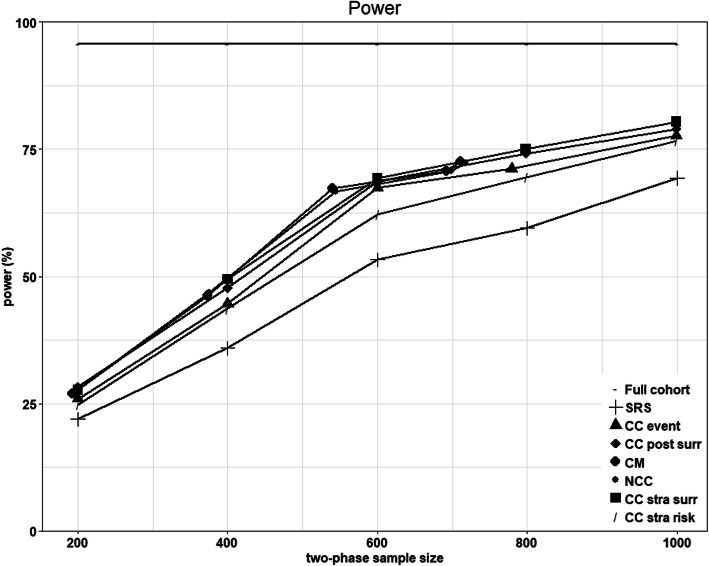
Power for different sample sizes of phase II (n). Scenario: censoring rate *ρ* = 0.4, common biomarker (25%), hazard ratio of biomarker = 1.5, sensitivity (i.e. probability of having a positive surrogate if the biomarker is positive) =0.7 and specificity (i.e. probability of having a negative surrogate if the biomarker is negative) = 0.7. Legend: CC stra surr (Case-Control stratified by surrogate), CC post surr (Case-Control post stratified by surrogate), CC event (Case-Control), CM (Counter-Matching) and SRS (simple random sampling)

In Fig. 4 we have explored the power by the size of the phase II sample. Up to a phase II sample size of nearly 500 individuals (1/4 of the entire cohort), CM and NCC were the most powerful designs. For larger sample sizes, CC stratified for the surrogate was the most powerful design. By note, both NCC and CM were sampled considering one control selected for each case and controls could be resampled, thus the sample size of phase II was constrained not to exceed twice the number of events in the entire cohort (thus it not always reached the planned sample size n).

### Application on the real data

The study that motivated our work was performed to evaluate the role of different genetic polymorphisms on treatment failure due to relapse [14, 15] and used data from a large Italian clinical trial (ClinicalTrials.gov identifier NCT00613457) [39]. Clinical data and other information were available for the whole trial cohort (phase I) of 1999 consecutive patients newly diagnosed with childhood acute lymphoblastic leukemia between 2000 and 2006. Biological samples were stored at diagnosis and were used to measure the genetic polymorphism of interest (phase II). In the study of Franca et al. [15] the subsample on which to measure the genetic polymorphism was chosen after classifying patients into six strata according to the event of interest (relapse/no relapse) and a three-level risk group stratification defined by prognostic features in the treatment protocol. Patients were sampled at random without replacement from each stratum, according to an optimal sampling strategy [40]. In particular, the sampling fractions for each stratum were chosen proportionally to the genetic variability reported within each of the strata to maximize the precision of the estimate of the genotype effect on the outcome. Of note, this was possible only due to the availability of pilot data on the genetic polymorphism of interest, that actually are not often available.

Overall, out of the 766 children for whom genotyping was required (approximately 1.5 controls for each case), the biomarker of interest (GST-θ) was obtained on 601 patients, getting a hazard ratio (HR) of 1.34 (95%CI: 0.90–2.00). By breaking up the variance of the coefficient of GST-θ into phase I and II contributions, we derived the efficiency of the design with respect to the expected one in the full cohort (estimate of the minimum irreducible uncertainty for the cohort) that resulted 54% by having genotyped 1/3 of the sample. Interestingly, the efficiency we got was higher than the expected one in any of the CC designs considered (see Table 2), as computed by simulations developed in this paper. Thus, the use of pilot data for an optimal sampling strategy compensated the lack of a surrogate variable.

**Table 2:** Efficiency (refers to the full cohort), design effect (refers to Simple Random Sampling) and power for SRS and Case-Control (CC) designs with hypothetical hazard ratio of the biomarker of interest (*HR*_*BM*_) of 1.3 and 1.5, biomarker common (25%), censoring rate *ρ* = 0.1, type I error 0.05

	SRS	Case-control	CC stratified by surrogate	CC stratified by risk factor
Efficiency				
***HR***_***BM***_ = 1.3	30.40%	38.91%	43.06%	34.47%
***HR***_***BM***_ = 1.5	25.98%	36.26%	38.51%	32.73%
Design effect				
***HR***_***BM***_ = 1.3	–	1.23	1.37	1.20
***HR***_***BM***_ = 1.5	–	1.22	1.36	1.18
Power				
***HR***_***BM***_= 1.3	30.91%	54.80%	60.15%	54.34%
***HR***_***BM***_= 1.5	58.35%	68.10%	70.65%	65.40%

Power evaluation was not done in this study at the design stage, but according to our simulations results (see Table 2), a sample size of *n* = 601 subjects would have reached a power of 55 and 68% to detect an HRBM of 1.3 and 1.5, respectively with a CC design. If CC stratified by surrogate would have been considered, an increase of power would have been obtained (60 and 71% respectively for an HRBM of 1.3 and 1.5), but still not reaching a reasonable value (i.e. 80%). This illustrates that being aware of power in the planning phase is very important.

## Discussion

This work underlines the importance of a careful study design in retrospective studies evaluating a new research question using available cohort data on which to measure additional characteristics, such as a new biomarker. The possibility to sample only a few controls and cases implies significant savings in cost and time and the evaluation of time-matching is also an important issue when the biomarker is affected by storage time or batch effects. We showed the advantages we can get in terms of efficiency and power by using available data and the importance of power evaluation in order to avoid useless studies. We also provide a tool to compute power by simulations (see the additional file for the R code).

From the simulation results, we found that the weighted Cox model provided valid estimates of biomarker effect and good coverage probabilities in the considered designs. The availability of auxiliary/surrogate variables of the biomarker of interest in phase I, the amount of censoring and the prevalence of the biomarker, together with power considerations could help researchers to identify the most efficient design. As expected, CC provided better efficiency with respect to SRS design, while PPS did not show much advantage [5]. If some covariates are expected to be associated with the new biomarker, it is advantageous to use them to define strata in a two-phase design, especially if they have a good accuracy in predicting its value and when the biomarker has low prevalence. Of note, simulation results showed that using these surrogate variables of the biomarker just in the analysis stage (and not as strata in the design) is also improving efficiency and power. Interestingly, if a variable is associated both with the biomarker and with the event of interest, such as the “confounder”, or just with the event of interest, such as the “risk factor”, using it to define strata did not show any advantage in power. Nevertheless, the inclusion of the “risk factor” in the Cox model is beneficial.

In the presence of censoring, sampling designs matching on time (NCC and CM) have shown higher performance in terms of design effect than CC and CC stratified by the surrogate designs, respectively. Similar results were found by Borgan and Olsen, that also suggested to combine the simple and counter-matching designs (sampling some controls by simple random sampling and others by stratified random sampling) [41]. Higher design effect is not always followed by an improvement in power as the last one depends also on the direction of bias that actually is favouring the CC design (as shown in Additional file Table S2). Moreover, matched designs are constrained to have a fixed integer number for the case/control ratio and this could result as a disadvantage in some settings. In the absence of censoring, results showed that CC is more powerful compared to the all other designs. Stoer and colleagues found similar results and called this particular condition as “CC extreme” design [42], as in this setting controls have the longest possible follow-up (subjects event-free at the end of follow-up in the absence of censoring). We also found, similarly to [22, 26], that CM has a marked efficiency advantage especially when the biomarker is rare, as surrogate information helps in sampling more subjects with the biomarker.

One limitation of our work is that we have considered only 1:1 matching ratio, but we did a fair evaluation by comparing the perfomance of different designs at the same sample size. Moreover, to emulate the ALL data, we have considered only a moderate effect of biomarker positivity on the event and we have assumed time-constant coefficients. However the general indications coming from our work are consistent with previous results across different settings, as well as for different specifications of the outcome model, as explored with sensitivity analyses. Moreover, the code developed, available at https://github.com/Fgraziano?tab=repositories, is helpful to investigate the power of different sampling designs in various setting.

## Conclusions

Summarizing, for efficient selection of the subcohort, we recommend the use of the information available on the entire cohort, as suggested in the flowchart of Additional file Figure S2 in supporting information. If a surrogate variable of the biomarker of interest is available, we suggest to use a case-control study stratified by the surrogate variable or a counter-matching design. The latter choice should be driven by the rate of censoring: if censoring is low we might opt for CC stratified, otherwise CM is more convenient. If the surrogate variable is not available, we should consider using CC or NCC as a design, depending on the censoring rate. As NCC and CM designs are constrained by a fixed ratio between cases and controls (1:1, 1:2 …), the overall sampling fraction with respect to the number of cases should be also considered together with power evaluation. In fact, if sufficient power would be reached with a phase II sample size n of nearly all cases plus a number of controls equal to 1.5 the number of cases, a CC design would be more convenient with respect to the matched designs. Moreover, we found that optimal sampling strategies using potentially available pilot data are greatly efficient. Thus, optimal sampling strategies for survival data would be very useful together with an user-friendly instrument to support researchers during the planning phase focusing on the choice of the stratification variable collected in phase I.

## Supplementary Information

**Additional file 1: Table S1.** Variables and parameters used in the simulation settings. **Figure S1.** Boxplots of *β*_*BM*_ estimates across 2000 replications for the scenario showed in Table 1a (upper panel) and 1b (lower panel). The left panel provides results for no censoring (*ρ* = 0), the middle panel for *ρ* = 0.1 and the right panel for *ρ* = 0.4. The solid line is the true value of biomarker effect estimate. Boxplots report minimum, maximum, and 3 quartiles values. Values that are far from the box by more than 1.5 times the interquartile range are reported by empty dots. Points in the boxplots are the mean values. **Table S2.** Beta estimates, length and coverage of CI 95% referred to sampling with *n* = 600 individuals, accuracy of surrogate: sensitivity = 0.7 and specificity = 0.7 and biomarker common (a) and rare (b). **Table S3.** Simulation results of the weighted Cox models adjusted for the confounder, *X*_*Conf*_, and risk factor, *X*_*Risk Fact*_, variables (a) and for the confounder, *X*_*Conf*_, risk factor, *X*_*Risk Fact*_, and surrogate, *X*_*Surr*_, variables (b). Scenario: fixed *n* = 600 individuals, accuracy of surrogate: sensitivity = 0.7 and specificity = 0.7, moderate censoring (censoring rate = 0.1) and common biomarker. **Table S4.** Design effect calculated using Mean Square Error refers to the same setting in Table 1 with sampling of 600 individuals, accuracy of surrogate: sensitivity = 0.7 and specificity = 0.7 and biomarker common (left) and rare (right). **Figure S2.** Proposal process flowchart to plan a sub-sampling from a cohort study. *if a surrogate of the biomarker is identified at the analysis stage, it is advantageous to post-stratify for it CC: Case-Control, CM: Counter-matching, NCC: Nested Case-Control. **File S1.** Some example R code to reproduce the results in Table 1.

## Data Availability

The simulation codes that support the findings of this study are openly available at https://github.com/Fgraziano?tab=repositories.

## Abbreviations

ALL: Acute lymphoblastic leukemia
CC: Case-control
CM: Counter-matching
HR: Hazard ratio
NCC: Nested case-control
SRS: Simple random sample
PPS: Probability proportional to size
