# Correction: Sampling strategies to evaluate the prognostic value of a new biomarker on a time-to-event end-point

**DOI:** 10.1186/s12874-022-01627-4

**Published:** 2022-06-01

**Authors:** Francesca Graziano, Maria Grazia Valsecchi, Paola Rebora

**Affiliations:** BICOCCA BIOINFORMATICS BIOSTATISTICS AND BIOIMAGING CENTRE-B4, School of Medicine and Surgery, University of Milano – Bicocca, Via Cadore 48, 20900 Monza, Italy

**Correction: BMC Med Res Methodol 21, 93 (2021)**

**https://doi.org/10.1186/s12874-021-01283-0**

Following publication of the original article [1], the authors noticed the two errors. The first one in the *Notation* *setting* sub-section of the *Methods* (in the PDF version this is at page 2, second column, lines 9-11).

The published version of the phrase is:

*Let π*_*i*_* = P(*$\xi_i$ * = 1|X*_*i*_*,Δ*_*i*_*,Z*_*i*_*) being the inclusion probability of subject i for the phase II sample, conditional on being selected at phase I.*

The correct version is given below:

*Let π*_*i*_ *= P(* $\xi_i$ *= 1|X*_*i*_ *,Δ*_*i*_ *), with Δ*_*i*_ *= I(T*_*i*_ *< C*_*i*_ *), being the inclusion probability of subject i for the phase II sample, conditional on being selected at phase I.*

A second error was found in the *Phase* *II* *sample* sub-section of the *Methods* (in the PDF version this is at page 3, first column, paragraph (iv)).

The published version of equation is:

$${\pi}_i=\frac{n/2}{\sum_{i=1}^N{\varDelta}_{\mathrm{i}}}$$

The correct version is given below:

$${\pi}_i=\frac{\sum_{i=1}^N{\varDelta}_i{\xi}_{{i}}}{\sum_{i=1}^N{\varDelta}_{{i}}}$$

The original article has been updated.
