# Supplementary material for: Sampling strategies to evaluate the prognostic value of a new biomarker on a time-to-event end-point
Source: BMC Med Res Methodol. 2021 Apr 30;21:93. doi: 10.1186/s12874-021-01283-0 (PMC8091513; doi:10.1186/s12874-021-01283-0)
Supplement: Supplementary file 1 — Additional file 1: Table S1. Variables and parameters used in the simulation settings. Figure S1. Boxplots of βBM estimates across 2000 replications for the scenario showed in Table 1a (upper panel) and 1b (lower panel). The left panel provides results for no censoring (ρ = 0), the middle panel for ρ = 0.1 and the right panel for ρ = 0.4. The solid line is the true value of biomarker effect estimate. Boxplots report minimum, maximum, and 3 quartiles values. Values that are far from the box by more than 1.5 times the interquartile range are reported by empty dots. Points in the boxplots are the mean values. Table S2. Beta estimates, length and coverage of CI 95% referred to sampling with n = 600 individuals, accuracy of surrogate: sensitivity = 0.7 and specificity = 0.7 and biomarker common (a) and rare (b). Table S3. Simulation results of the weighted Cox models adjusted for the confounder, XConf, and risk factor, XRisk Fact, variables (a) and for the confounder, XConf, risk factor, XRisk Fact, and surrogate, XSurr, variables (b). Scenario: fixed n = 600 individuals, accuracy of surrogate: sensitivity = 0.7 and specificity = 0.7, moderate censoring (censoring rate = 0.1) and common biomarker. Table S4. Design effect calculated using Mean Square Error refers to the same setting in Table 1 with sampling of 600 individuals, accuracy of surrogate: sensitivity = 0.7 and specificity = 0.7 and biomarker common (left) and rare (right). Figure S2. Proposal process flowchart to plan a sub-sampling from a cohort study. *if a surrogate of the biomarker is identified at the analysis stage, it is advantageous to post-stratify for it CC: Case-Control, CM: Counter-matching, NCC: Nested Case-Control. File S1. Some example R code to reproduce the results in Table 1. [file 12874_2021_1283_MOESM1_ESM.pdf]

Additional file for:

Sampling strategies to evaluate the prognostic value of a new biomarker on a time-to-event end-point

by Francesca Graziano, Maria Grazia Valsecchi and Paola Rebora

Table S1. Variables and parameters used in the simulation settings

Variable	Probability (P)	Frequency/accuracy	parameters	Coefficient (β)	Hazard Ratio (HR)
X_{Conf}	$P(X_{Conf}=1)=0.5$			0.23	1.26
X_{BM}	$P(X_{BM}=1\|X_{Conf}) = \frac{\exp(a+b*X_{conf})}{1+\exp(a+b*X_{conf})}$	Common with frequency~ 25%	a=-2, b=1.7	0.40	1.5
		Rare with frequency ~ 5%	a=-4, b=1.5		
X_{Surr}	$P(X_{Surr}=1\|X_{BM}) = \frac{\exp(c+d*X_{BM})}{1+\exp(c+d*X_{BM})}$	Specificity=70%, Sensitivity=70%;	c=-0.87, d=1.74	0	1
		Specificity=70%, Sensitivity=80%;	c= -0.9, d=2.6		
		Specificity=70%, Sensitivity=90%;	c= -1.1, d=3.3		
		Specificity=90%, Sensitivity=70%;	c=-2.5 d=3.3		
		Specificity=90%, Sensitivity=90%;	c=-2.4, d=4.5		
$X_{Risk\ Factor}$	$P(X_{Risk\ factor}=1)=0.4$			0.28	1.32

Figure S1. Boxplots of β_{BM} estimates across 2000 replications for the scenario showed in Table 1a (upper panel) and 1b (lower panel). The left panel provides results for no censoring (ρ = 0), the middle panel for ρ = 0.1 and the right panel for ρ = 0.4. The solid line is the true value of biomarker effect estimate. Boxplots report minimum, maximum, and 3 quartiles values. Values that are far from the box by more than 1.5 times the interquartile range are reported by empty dots. Points in the boxplots are the mean values.

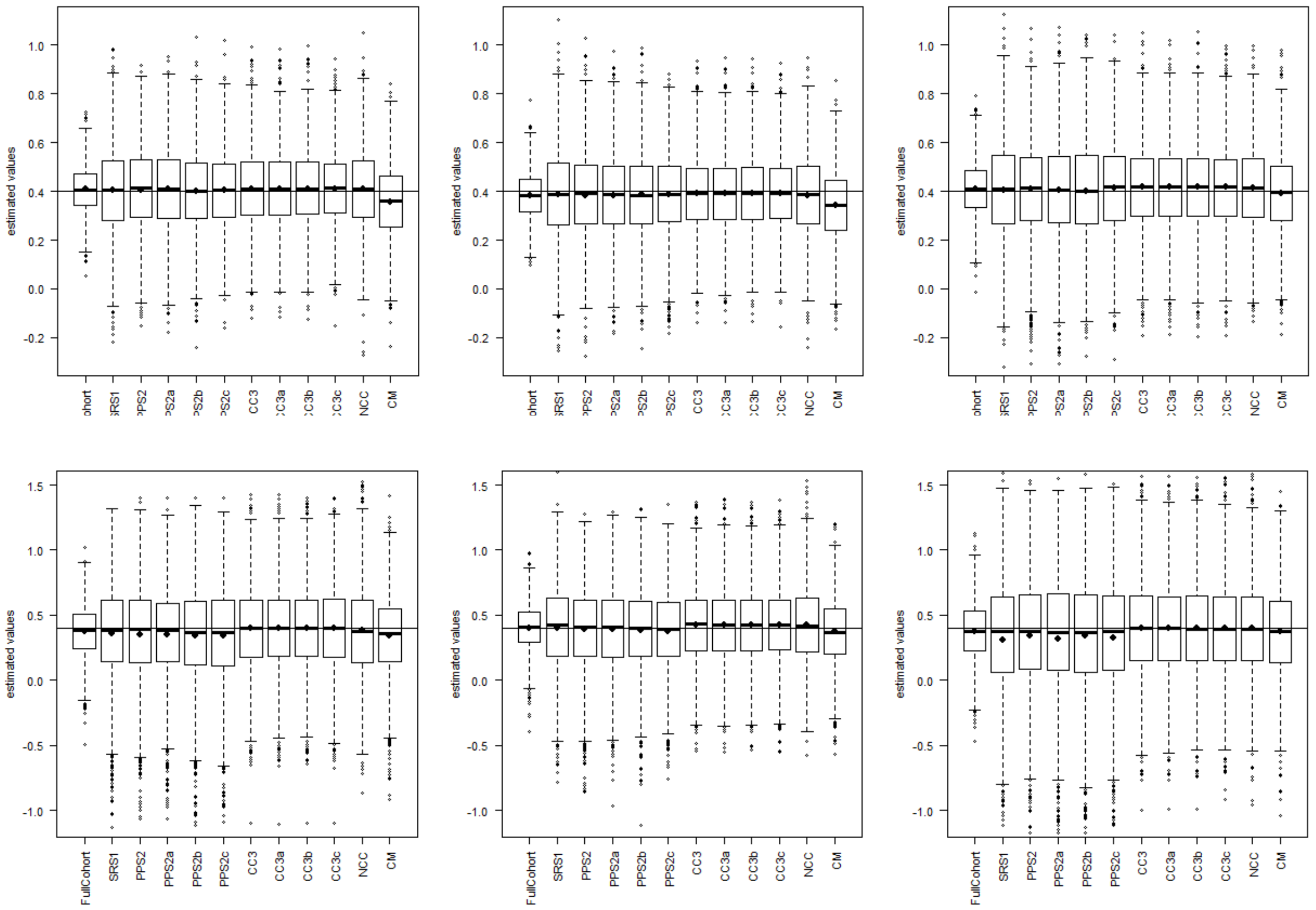

Table S2. Beta estimates, length and coverage of CI 95% referred to sampling with n=600 individuals, accuracy of surrogate: sensitivity = 0.7 and specificity = 0.7 and biomarker common (a) and rare (b)

**a) SAMPLING DESIGN**	**Stratification Variable**	$\hat{\beta}_{BM}$			**95%CI length**			**95%CI COVERAGE**		
		**Censoring Rate**			**Censoring Rate**			**Censoring Rate**		
		**0**	**0.1**	**0.4**	**0**	**0.1**	**0.4**	**0**	**0.1**	**0.4**
**Full cohort**	-	0.408	0.385	0.410	0.557	0.566	0.661	0.948	0.953	0.953
**1. SRS**	-	0.404	0.387	0.406	1.040	1.066	1.245	0.936	0.940	0.946
**2. PPS**	Event	0.407	0.385	0.407	1.042	1.063	1.246	0.947	0.949	0.949
**2a. PPS**	Event; Risk factor	0.408	0.384	0.404	1.044	1.061	1.244	0.948	0.951	0.947
**2b. PPS**	Event; Confounder	0.403	0.385	0.403	1.040	1.063	1.244	0.947	0.948	0.958
**2c. PPS**	Event; Surrogate	0.407	0.387	0.413	0.989	1.000	1.191	0.953	0.945	0.955
**3. CC**	Event	0.411	0.392	0.420	0.962	0.965	1.079	0.948	0.956	0.947
**3a. CC**	Event; Risk factor	0.410	0.390	0.408	0.979	0.983	1.085	0.946	0.952	0.948
**3b. CC**	Event; Confounder	0.412	0.385	0.410	0.962	0.972	1.067	0.946	0.954	0.947
**3c. CC**	Event; Surrogate	0.408	0.384	0.412	0.898	0.903	1.013	0.948	0.949	0.943
**4. NCC**	Event	0.408	0.382	0.414	1.013	0.989	1.062	0.953	0.950	0.946
**5. CM**	Event; Surrogate	0.356	0.342	0.391	0.839	0.855	0.978	0.927	0.936	0.956

**b) SAMPLING DESIGN**	**Stratification Variable**	$\hat{\beta}_{BM}$			**95%CI length**			**95%CI COVERAGE**		
		**Censoring Rate**			**Censoring Rate**			**Censoring Rate**		
		**0**	**0.1**	**0.4**	**0**	**0.1**	**0.4**	**0**	**0.1**	**0.4**
**Full cohort**	-	0.372	0.396	0.377	1.114	1.188	1.331	0.951	0.954	0.954
**1. SRS**	-	0.356	0.359	0.307	2.214	2.358	2.692	0.943	0.952	0.959
**2. PPS**	Event	0.349	0.377	0.340	2.213	2.363	2.719	0.961	0.946	0.959
**2a. PPS**	Event; Risk factor	0.352	0.360	0.319	2.210	2.352	2.690	0.957	0.957	0.950
**2b. PPS**	Event; Confounder	0.342	0.367	0.340	2.202	2.369	2.713	0.954	0.945	0.952
**2c. PPS**	Event; Surrogate	0.345	0.378	0.327	2.192	2.341	2.674	0.947	0.950	0.954
**3. CC**	Event	0.397	0.421	0.403	2.122	2.255	2.425	0.945	0.952	0.940
**3a. CC**	Event; Risk factor	0.397	0.415	0.406	2.124	2.288	2.537	0.946	0.936	0.944
**3b. CC**	Event; Confounder	0.397	0.424	0.412	2.127	2.251	2.437	0.945	0.943	0.947
**3c. CC**	Event; Surrogate	0.398	0.411	0.390	2.113	1.968	2.154	0.948	0.939	0.939
**4. NCC**	Event	0.380	0.424	0.399	2.255	2.392	2.423	0.949	0.944	0.945
**5. CM**	Event; Surrogate	0.343	0.358	0.371	1.776	1.829	2.065	0.938	0.934	0.944

Legend: CI-confidence interval; SRS–Simple Random Sample; PPS- Probability Proportional to size; CC- Case-Control;NCC- Nested Case-Control; CM- Counter-matching

Table S3. Simulation results of the weighted Cox models adjusted for the confounder, X_{Conf}, and risk factor, $X_{Risk\ Fact}$, variables (a) and for the confounder, X_{Conf}, risk factor, $X_{Risk\ Fact}$, and surrogate, X_{Surr}, variables (b). Scenario: fixed n=600 individuals, accuracy of surrogate: sensitivity = 0.7 and specificity = 0.7, moderate censoring (censoring rate =0.1) and common biomarker.

a) $h_i(t) = h_0(t)exp^{\beta_{BM}X_{BM_i}+\beta_{Conf}X_{Conf_i}+\beta_{Risk\ Fact}X_{Risk\ Fact_i}}$

SAMPLING DESIGN	Stratification Variable	BIAS	SE EMPIRICAL	MSE	95%CI COVERAGE	95%CI length	POWER (%)	DESIGN EFFECT
**Full cohort**	-	-0.003	0.098	0.009	0.942	0.569	98	-
**1. SRS**	-	-0.008	0.181	0.033	0.949	1.066	59	-
**2. PPS**	Event	-0.005	0.183	0.033	0.943	1.070	61	1.004
**2a. PPS**	Event; Risk factor	-0.000	0.179	0.032	0.945	1.074	61	1.005
**2b. PPS**	Event; Confounder	-0.005	0.185	0.034	0.943	1.070	59	1.005
**2c. PPS**	Event; Surrogate	-0.006	0.172	0.029	0.95	1.011	64	1.116
**3. CC**	Event	0.002	0.164	0.026	0.945	0.973	69	1.218
**3a. CC**	Event; Risk factor	0.002	0.162	0.026	0.952	0.972	69	1.218
**3b. CC**	Event; Confounder	-0.002	0.165	0.027	0.938	0.972	67	1.210
**3c. CC**	Event; Surrogate	-0.002	0.152	0.023	0.955	0.913	74	1.362
**4. NCC**	Event	-0.007	0.167	0.027	0.95	0.997	65	1.142
**5. CM**	Event; Surrogate	-0.049	0.148	0.024	0.941	0.852	65	1.421

b) $h_i(t) = h_0(t)exp^{\beta_{BM}X_{BM_i}+\beta_{Conf}X_{Conf_i}+\beta_{Risk\ Fact}X_{Risk\ Fact_i}+\beta_{Surr}X_{Surr_i}}$

SAMPLIN G DESIGN	Stratification Variable	BIAS	SE EMPIRICAL	MSE	95%CI COVERAGE	95%CI length	POWER (%)	DESIGN EFFECT
**Full cohort**	-	-0.004	0.106	0.011	0.948	0.623	96	-
**1. SRS**	-	-0.009	0.195	0.038	0.951	1.1770	52	-
**2. PPS**	Event	-0.004	0.200	0.040	0.944	1.183	52	1.007
**2a. PPS**	Event; Risk factor	-0.002	0.200	0.040	0.944	1.186	53	1.007
**2b. PPS**	Event; Confounder	-0.005	0.203	0.041	0.934	1.182	52	1.008
**2c. PPS**	Event; Surrogate	-0.006	0.198	0.039	0.945	1.182	53	1.007
**3. CC**	Event	0.001	0.175	0.031	0.954	1.071	62	1.223
**3a. CC**	Event; Risk factor	0.001	0.176	0.031	0.952	1.073	61	1.219
**3b. CC**	Event; Confounder	0.001	0.175	0.031	0.952	1.073	62	1.219
**3c. CC**	Event; Surrogate	0.001	0.176	0.030	0.952	1.073	61	1.219
**4. NCC**	Event	-0.007	0.184	0.033	0.946	1.099	57	1.148
**5. CM**	Event; Surrogate	-0.040	0.164	0.028	0.945	0.955	58	1.409

Legend: SE-Standard Error; MSE-Mean square Error; CI-confidence interval; SRS–Simple Random Sample; PPS-Probability Proportional to size; CC- Case-Control;NCC- Nested Case-Control; CM- Counter-matching

Table S4 – Design effect calculated using Mean Square Error refers to the same setting in Table 1 with sampling of 600 individuals, accuracy of surrogate: sensitivity = 0.7 and specificity = 0.7 and biomarker common (left) and rare (right).

		**BIOMARKER**					
		**COMMON (~25%)**			**RARE (~5%)**		
		**Censoring Rate**			**Censoring Rate**		
**SAMPLING DESIGN**	**Stratification Variable**	**0**	**0.1**	**0.4**	**0**	**0.1**	**0.4**
**2. PPS**	Event	1.102	1.068	1.062	0.974	0.990	0.883
**2a. PPS**	Event; Risk factor	1.108	1.135	1.008	1.021	1.084	0.952
**2b. PPS**	Event; Confounder	1.088	1.083	1.028	0.966	1.034	0.342
**2c. PPS**	Event; Surrogate	1.271	1.187	1.166	0.935	1.145	0.944
**3. CC**	Event	1.301	1.396	1.313	1.305	1.289	1.322
**3a. CC**	Event; Risk factor	1.253	1.260	1.282	1.207	1.283	1.381
**3b. CC**	Event; Confounder	1.259	1.340	1.388	1.331	1.301	1.336
**3c. CC**	Event; Surrogate	1.503	1.465	1.458	1.482	1.680	1.552
**4. NCC**	Event	1.153	1.261	1.377	1.140	1.206	1.441
**5. CM**	Event; Surrogate	1.308	1.288	1.537	1.432	1.671	1.583

Figure S2. Proposal process flowchart to plan a sub-sampling from a cohort study.

*if a surrogate of the biomarker is identified at the analysis stage, it is advantageous to post-stratify for it

CC: Case-Control, CM: Counter-matching, NCC: Nested Case-Control.

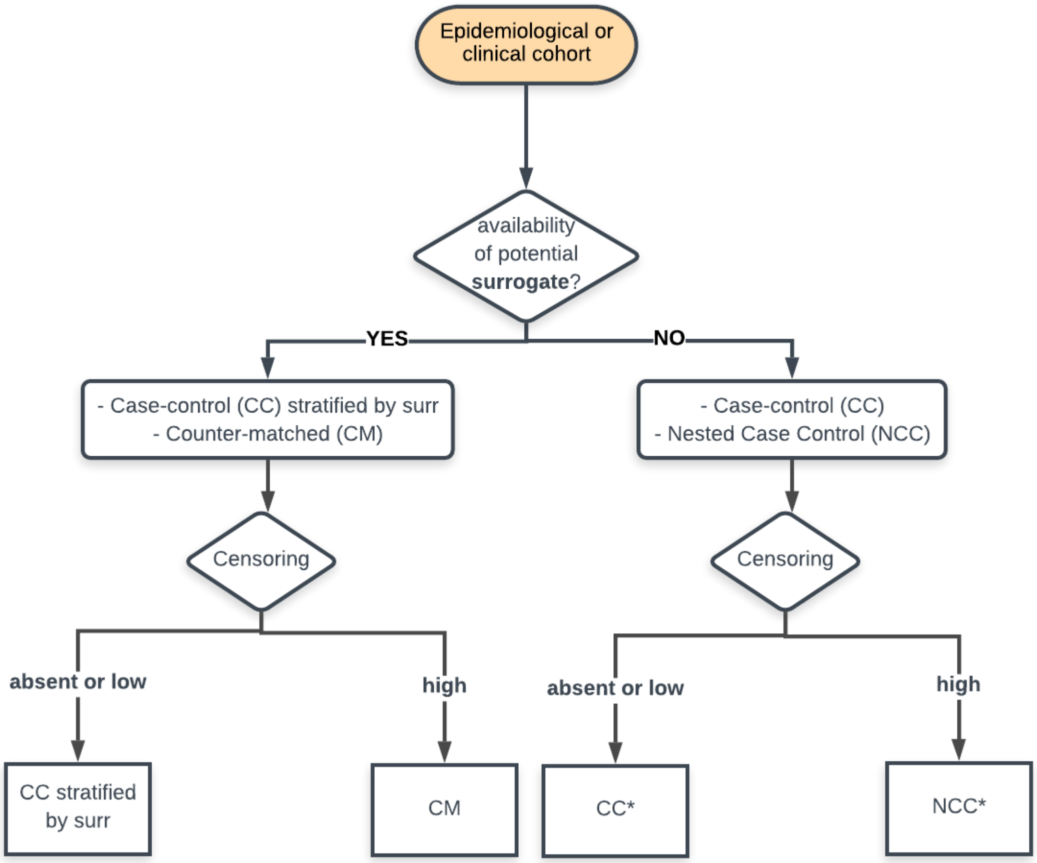

File S1. Some example R code to reproduce the results in Table 1

Considering the scenario where the dataframe has a common biomarker and sensitivity and sensibility of the surrogate set at 70%. To generate the matrix of covariates `X` including the biomarker value (X_{BM}), the risk factor ($X_{Risk\ Factor}$), and the confounder (X_{Conf}) we perform the following R code:

```
#confounder
x4   <-  rbinom(N, 1, prob=0.50)
#biomarker
a<--2
b<-1.7
x1 <- rbinom(N, 1, prob=exp(a+b*x4)/(1+exp(a+b*x4)))
#risk factor
x2 <-  rbinom(N, 1, prob=0.40)
#surrogate
c<--0.87
d<-1.74
x3 <-  rbinom(N, 1, prob=exp(c+d*x1)/(1+exp(c+d*x1)))

# beta's coefficients
beta1<-BETA<-0.40
beta2 <- 0.28
beta3<-0
beta4<- 0.23

beta<-c(beta1,beta2,beta3,beta4)
X<-cbind(x1,x2,x3,x4)
```

Obviously, to achieve the other scenarios reported in the paper, we simulated `X` using the parameters in concordance with the values reported in Table S1.
Then, we use the following function accessible on github (https://github.com/Fgraziano/SimulationCode/blob/master/SimulationCode.R) to calculate power and other performance measures. For scenario:

I) with no censoring:

```
simul(lambda=0.1, k=0.9,  beta=0.4,X=X,
rate=0,follow=2,N=2000,n=600,B=2000,ssed=123)
```

II) censoring = 0.1:

```
simul(lambda=0.1, k=0.9,  beta=0.4,X=X,
rate=0.1,follow=2,N=2000,n=600,B=2000,ssed=123)
```

III) censoring = 0.4:

```
simul(lambda=0.1, k=0.9,  beta=0.4,X=X,
rate=0.4,follow=2,N=2000,n=600,B=2000,ssed=123)
```
